# Regional Burden of Urinary Tract Infection, Its Aetiologies, and Antibiotic Resistance Patterns, 2020–2023: A Retrospective Study

**DOI:** 10.1002/hsr2.71503

**Published:** 2025-11-11

**Authors:** Solomon Wireko, Gifty Ngmaakan, Doreen Dwomoh, Charlotte Hansen, Sampson Nartey, Ebenezer Senu

**Affiliations:** ^1^ Deapartment of Laboratory Technology, Faculty of Health Sciences Kumasi Technical University Kumasi Ghana; ^2^ Public Health and Reference Laboratory Ghana Health Service Kumasi Ghana; ^3^ Department of Medical Diagnostics Kwame Nkrumah University of Science and Technology Kumasi Ghana

**Keywords:** antibiogram, antimicrobial resistance, bacteriuria, type 2 diabetes mellitus (T2DM), urinary tract infections

## Abstract

**Background and Aims:**

The emergence of antibiotic‐resistant strains of urinary tract infections (UTI) pathogens poses a significant challenge to the effective treatment of this condition. This study aimed to conduct a retrospective study to analyze the etiology and antibiotic resistance patterns of common urinary tract pathogens in the Ashanti Region of Ghana.

**Methods:**

The study employed a standardized data collection form to extract information on age, sex, urine culture results, antibiotic susceptibility test results, and antibiotic treatment regimens from 2020 to 2023.

**Results:**

The primary agent responsible for UTIs was *Escherichia coli*, identified in 47.8% of cases, followed by *Klebsiella pneumoniae* at 15.8%, *Staphylococcus aureus* at 9.2%, and *Pseudomonas aeruginosa* at 6.5%. *Candida* species were detected in 12% of cases, while coagulase‐negative Staphylococci constituted 3.8%. *Proteus* and *Providencia* species were observed in 1.1% and 0.5%, respectively. *E. coli* exhibited complete resistance (100%) to Nalidixic acid (NAL) and Ofloxacin (OFL), with varying degrees of resistance observed across other antibiotics. Among the 29 isolates of *K. pneumoniae*, 90.0% showed resistance against Cefotaxime, 81.3% against Ciprofloxacin, and 80% against Trimethoprim/Sulfamethoxazole. Sixty percent of *E. coli* isolates and 25% of *Klebsiella* isolates demonstrated multi‐drug resistance and ESBL production.

**Conclusion:**

The surge in multidrug‐resistant (MDR) gram‐negative isolates presents a significant healthcare hurdle for UTI patients. A noteworthy portion of prevalent pathogens, resistant to NAL and OFL, falls into the category of ESBL‐producing *Enterobacterales* and *Pseudomonas* spp. The limited availability of effective treatments for MDR gram‐negative isolates underscores the urgent need for innovative therapeutic alternatives.

## 1 Introduction

Urinary tract infections (UTIs) represent a prevalent global health concern, affecting millions of individuals annually and posing a substantial burden on healthcare systems [1, 2, 3]. Defined by the presence of pathogenic microorganisms in the urinary system, UTIs encompass a spectrum of conditions ranging from asymptomatic bacteriuria to severe, recurrent infections [4, 5, 6]. Common microbial agents of UTI include *Escherichia coli*, *Klebsiella pneumoniae*, *Enterococcus faecalis*, and *Pseudomonas aeruginosa*. Among the diverse demographic groups affected, individuals with compromised immune systems or underlying medical conditions, such as type 2 diabetes mellitus (T2DM) patients, are particularly vulnerable to urinary tract complications [7, 8, 9]. Aging, immunosuppressive therapy, chronic kidney disease, and malignancies are additional causes of immune compromise, alongside T2DM.

Although there is limited data on the overall national occurrence of UTIs in Ghana, two investigations have indicated a prevalence of 42.75% in pregnant women [10] and 86% in hospitalized adults [11]. Reports suggest that the percentage of multidrug‐resistant (MDR) UTIs in Ghana can be as high as 93.6% [12, 13].

Understanding the regional variations in UTI prevalence, causative agents, and antibiotic resistance patterns is crucial for informed public health interventions. This retrospective study aims to analyze the antibiotic resistance patterns of common urinary tract pathogens spanning the years 2020 to 2023. By examining a comprehensive data set, we seek to elucidate the magnitude of the UTI burden, identify the prevalent aetiologies, and unravel the evolving patterns of antibiotic resistance within our defined geographical scope.

Several studies have shed light on the global impact of UTIs, emphasizing the need for context‐specific investigations to address regional subtle variations or differences [14, 15]. Moreover, the intersectionality of UTIs with chronic conditions like T2DM adds layers of complexity, demanding the ability to grasp and appreciate the fine details, exceptions, and context‐specific elements that contribute to a more comprehensive and insightful perspective on the interplay between host factors, regional epidemiology, and microbial characteristics [4, 16]. This study is motivated by the need to fill gaps in the existing literature, providing a detailed analysis of the regional dynamics surrounding UTIs.

## 2 Materials and Methods

### 2.1 Study Design and Site

The study adopted a retrospective design, encompassing data collected over the period from 2020 to 2023. This design involved the analysis of historical patient records, laboratory reports, and relevant documentation to investigate UTIs within the specified timeframe. The data were collected from five healthcare facilities, including one tertiary hospital and four district hospitals within the Ashanti Region. The retrospective approach allows for the examination of trends, prevalence rates, causative agents, and antibiotic resistance patterns over the 3‐year period. The study was focused on the Ashanti region of Ghana, and the analysis is tailored to understand the dynamics of UTIs within this defined area. The study included both inpatient and outpatient cases. Approximately 34.8% (64/184) of the samples were from outpatient departments, and 65.2% (120/184) were from hospitalized patients.

### 2.2 Data Collection

The primary sources of data for this study included electronic health records (EHRs), laboratory reports detailing diagnostic findings related to UTIs, and patient charts containing relevant clinical details.

Information extracted from EHRs encompassed demographic data, medical history, laboratory test results, and prescribed treatments. This comprehensive data set allowed for a thorough analysis of patient profiles and UTI‐related information. Laboratory reports contributed essential microbiological data, identifying causative agents of UTIs, and offered insights into bacterial isolates, antibiotic susceptibility patterns, and any additional relevant diagnostic information.

Detailed patient charts also provided clinicians' notes, treatment regimens, and any pertinent clinical observations. These records contributed to a holistic understanding of individual patient cases.

### 2.3 Antimicrobial Susceptibility Testing

Information on antimicrobial susceptibility patterns was extracted from laboratory records of urine culture reports between 2020 and 2023. According to standard protocol at the participating laboratories, susceptibility testing was performed using the Kirby‐Bauer disk diffusion method, and results were interpreted following the Clinical and Laboratory Standards Institute (CLSI) 2020 guidelines. Only isolates with complete susceptibility data were included in the analysis. ESBL production was detected using the phenotypic confirmatory combination disk method, following CLSI guidelines.

MDR was defined according to the international consensus by Magiorakos et al. [17] as resistance to at least one agent in three or more antimicrobial categories.

### 2.4 Inclusion and Exclusion Criteria

Patients included in the study were those with a documented diagnosis of UTI during the specified period from 2020 to 2023. This timeframe ensures a contemporary representation of UTI cases and allows for the exploration of potential temporal trends. Emphasis was placed on capturing a diverse range of UTI cases, considering variations in severity, recurrence, and comorbidities.

Patients with incomplete or insufficient data (records missing key clinical information such as urine culture results, antibiotic susceptibility profiles, or patient demographic details) hindering a comprehensive analysis were excluded, as well as cases involving UTIs with known alternative aetiologies, such as urinary tract abnormalities or malignancies, to maintain the specificity of the study.

### 2.5 Ethical Considerations

The study received ethical approval (IRID/EC2022/HS003) from the Institute of Research Innovation and Development (IRID) of Kumasi Technical University, and the committee waived the requirement for informed consent due to the retrospective and anonymized nature of the study. Permission was granted by the Ghana Health Service Ashanti Regional Health Directorate (Ref. No. GHS/ASH/INTRO).

### 2.6 Data Access and Usage

The study strictly adhered to ethical standards regarding data access and usage. Access to patient records and related information was granted only to authorized personnel involved in the research by the Ashanti Regional Health Directorate of the Ghana Health Service, and all data was handled in compliance with institutional and legal regulations. All data collected, including EHRs, laboratory reports, and patient charts, were treated with the utmost confidentiality. Personal identifiers were appropriately anonymized to prevent the disclosure of individual identities.

### 2.7 Data Analysis

Data was cleaned and exported to the Statistical Package for the Social Sciences (IBM SPSS Statistics version 26). The data were expressed in percentages and frequencies, and the Chi‐square test was used to determine significant differences between the variables. Statistical significance was accepted in all comparisons at a *p‐*value less than 0.05.

## 3 Results

### 3.1 Age and Gender Distribution of Study Participants With Positive Bacterial Infection

Of the 184 individuals diagnosed with positive bacterial infections, the preponderance consisted of females, comprising 70.1% (129/184) of the total, with males representing 29.9% (74/184). Most participants were distributed across the age brackets of 21–40 and 41–60 years, constituting 40.8% (75/184) and 25.5% (46/184) of the overall participant count, respectively. The age range of 1–20 years constituted 12% (22/184) of the participants, while individuals aged 61–80 and 81–100 years comprised 17.4% (32/184) and 4.3% (8/184) of the study cohort (see Table 1).

**Table 1:** Age and gender distribution of study participants with positive bacterial infection.

Variable	Total (*n* = 184)	Percentage (%)
Age		
1–20	22	12
21–40	75	40.8
41–60	47	25.5
61–80	32	17.4
81–100	8	4.3
Gender		
Females	129	70.1
Males	55	29.9

### 3.2 Prevalence of Organisms Causing UTI Among Study Participants

**Figure 1:**
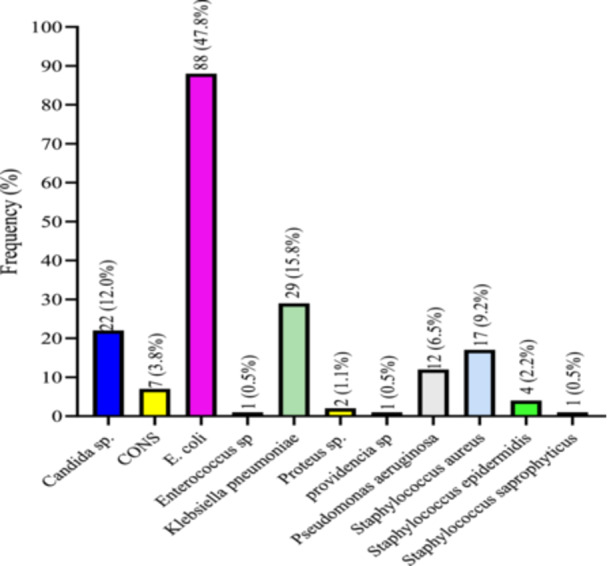
Prevalence of Organisms causing UTI among study participants.

*E. coli* was the most frequently isolated organism, accounting for 47.8% (88/155) of cases, followed by *K. pneumoniae* 15.8% (24/155), *Staphylococcus aureus* 9.2% (14/155), and *P. aeruginosa* 6.5% (10/155). *Candida* species were identified in 12% (19/155) of cases. Among the coagulase‐negative Staphylococci (CONS), *Staphylococcus epidermidis* accounted for 2.2%, while other CONS (excluding *Staphylococcus saprophyticus*) constituted 3.8% of isolates. *Proteus* and *Providencia* species were detected in 1.1% and 0.5% of cases, respectively. Both *Enterococcus* spp. and *S. saprophyticus* each represented 0.5% of the total isolates (Figure 1).

### 3.3 Age Distribution of Isolated Organisms Causing UTI Among Study Participants

This study did not identify any statistically significant association between the distribution of isolates and age groups. Notably, *E. coli* emerged as the predominant pathogen, particularly prevalent in the 41–60 years age group, constituting 57.4% of cases. *Candida* spp. cases were predominantly observed in the 1–20 age group, accounting for 22.7%. *Candida* isolates were reported only when found in significant colony counts (> 10⁵ CFU/mL) and in symptomatic patients, following standard diagnostic criteria for fungal UTIs. CONS and *Enterococcus* spp. were distributed across age groups, with CONS being most prevalent in the 1–20 age group at 9.1%. *Providencia* spp. was isolated exclusively in the 21–40‐year‐old age group. *K. pneumoniae* displayed varied prevalence, with the highest proportion in the 61–80 years age group at 21.9%. *Proteus* sp. and *P. aeruginosa* were primarily present in the 21–40 and 41–60 years age groups, with *P. aeruginosa* having the highest occurrence in the 81–100 age group at 25.0%. Additionally, *S. aureus* was detected across all age groups, with the highest proportion in the 1–20 years age group at 18.2%. *S. epidermidis* was mainly isolated in the 1–20 and 21–40 years age groups. Furthermore, *S. saprophyticus* was most commonly found in the 1–20 and 21–40 age groups (refer to Table 2).

**Table 2:** Age distribution of isolated organisms causing UTI among study participants.

		Age (years)					
	Total (*n* = 148)	1–20 (*n* = 22)	21–40 (*n* = 75)	41–60 (*n* = 47)	61–80 (*n* = 32)	81–100 (*n* = 8)	*p‐*value
Isolates							0.453
*Candida* sp.	22 (12.0)	5 (22.7)	11 (14.7)	4 (8.5)	2 (6.3)	0 (0.0)	
CONS	7 (3.8)	2 (9.1)	3 (4.0)	2 (4.3)	0 (0.0)	0 (0.0)	
*Escherichia coli*	88 (47.8)	5 (22.7)	32 (42.7)	27 (57.4)	20 (62.5)	4 (50.0)	
*Enterococcus* sp.	1 (0.5)	0 (0.0)	1 (1.3)	0 (0.0)	0 (0.0)	0 (0.0)	
*Klebsiella pneumoniae*	29 (15.8)	5 (22.7)	9 (12.0)	7 (14.9)	7 (21.9)	1 (12.5)	
*Proteus* sp.	2 (1.1)	0 (0.0)	1 (1.3)	1 (2.1)	0 (0.0)	0 (0.0)	
*Providencia* sp.	1 (0.5)	0 (0.0)	1 (1.3)	0 (0.0)	0 (0.0)	0 (0.0)	
*Pseudomonas aeruginosa*	12 (6.5)	0 (0.0)	4 (5.3)	4 (6.5)	2 (6.3)	2 (25.0)	
*Staphylococcus aureus*	17 (9.2)	4 (18.2)	10 (13.3)	2 (4.3)	1 (3.1)	0 (0.0)	
*Staphylococcus epidermidis*	4 (2.2)	1 (4.5)	2 (2.7)	0 (0.0)	0 (0.0)	1 (12.5)	
*Staphylococcus saprophyticus*	1 (0.5)	0 (0.0)	1 (1.3)	0 (0.0)	0 (0.0)	0 (0.0)	

### 3.4 Gender Distribution of Isolated Organisms Causing UTI Among Study Participants

This study identified a notable correlation between isolated organisms and gender (*p* < 0.0001). *Candida* sp. was predominantly found in females, constituting 17.1%, with no occurrences in males. *E. coli*, the most prevalent pathogen, was observed in both genders, with a majority of cases in females (51.2%) compared to males (40.0%). Similarly, CONS were more frequently isolated in females (4.7%) than in males (1.8%). *P. aeruginosa* exhibited a marked gender difference, with a minority of cases in females (2.3%) but a considerably higher prevalence in males (16.4%). *K. pneumoniae* was present in both genders, with a slightly higher proportion in males (20.0%) compared to females (14.0%). Additionally, *Proteus* sp. and *Providencia* sp. were identified in both genders, with a minor gender‐based variation. *Proteus* sp. was slightly more prevalent in males (1.8%) than in females (0.8%), while *Providencia* sp. was isolated in females (0.8%) but not in males. Furthermore, *S. aureus* was detected in both genders, with a slightly higher prevalence in males (12.7%) compared to females (7.8%). *S. epidermidis* exclusively appeared in males (7.3%), while *S. saprophyticus* was identified in females (0.8%) and absent in males (see Table 3).

**Table 3:** Gender distribution of Isolated Organisms causing UTI among study participants.

	Total (*n* = 148)	Females (*n* = 129)	Male (*n* = 55)	*p‐*value
Isolates				**< 0.0001**
*Candida* sp.	22 (12.0)	22 (17.1)	0 (0.0)	
CONS	7 (3.8)	6 (4.7)	1 (1.8)	
*Escherichia coli*	88 (47.8)	66 (51.2)	22 (40.0)	
*Enterococcus* sp.	1 (0.5)	1 (0.8)	0 (0.0)	
*Klebsiella pneumoniae*	29 (15.8)	18 (14.0)	11 (20.0)	
*Proteus* sp.	2 (1.1)	1 (0.8)	1 (1.8)	
*Providencia* sp.	1 (0.5)	1 (0.8)	0 (0.0)	
*Pseudomonas aeruginosa*	12 (6.5)	3 (2.3)	9 (16.4)	
*Staphylococcus aureus*	17 (9.2)	10 (7.8)	7 (12.7)	
*Staphylococcus epidermidis*	4 (2.2)	0 (0.0)	4 (7.3)	
*Staphylococcus saprophyticus*	1 (0.5)	1 (0.8)	0 (0.0)	

*Note:* Data presented as frequencies and percentages, *p*‐value computed by Chi‐square test, *p*‐value < 0.05, and bolded means statistically significant.

### 3.5 Antibiogram of Bacteria Isolated in This Study

A total of 19 antibiotics were assessed against various bacterial isolates, encompassing Aminoglycosides (Amikacin [AMI], Gentamicin [GEN]), Fluoroquinolones (Ciprofloxacin [CIP], Ofloxacin [OFL]), Sulfonamides (Trimethoprim/Sulfamethoxazole [SXT]), First Generation Cephalosporins (Cefuroxime [CRX]), Second Generation Cephalosporins (Cefotaxime [CTX]), Third Generation Cephalosporins (Ceftriaxone [CTR], Ceftazidime [CTZ]), Carbapenems (Meropenem [MEM]), Quinolones (Nalidixic Acid [NAL]), Tetracyclines (Doxycycline [DOX], Tetracycline [TET]), Penicillins (Amoxicillin [AMO], Ampicillin [AMP]), Macrolides (Azithromycin [AZI], Erythromycin [ERY]), Vancomycin (VAN), and Chloramphenicol (CMP).

For the seven CONS isolates, 100% resistance was noted against NAL and CMP, followed by a 33.3% resistance against AZI. *E. coli* exhibited 100% resistance against NAL and OFL, with varying proportions of resistance observed across all other antibiotics, while VAN was the only drug to which *E. coli* showed 100% sensitivity. Additionally, the single *Enterococcus* spp. isolate demonstrated 100% resistance to CIP.

Among the 29 *K. pneumoniae* isolates, substantial proportions were resistant to ERY (100.0%), CTX (90.0%), CIP (81.3%), SXT (80%), CRX (77.8%), AMO (66.7%), TET (66.7%), CMP (50%), and AMP (50%). The two *Proteus* sp. isolates exhibited 100% resistance to CIP, AZT, AMI, and MEM, and 50% resistance to CTR. However, *Proteus* sp. showed no resistance to SXT, AMO, and AZI (refer to Table 4).

**Table 4:** Antibiogram of bacteria isolated in this study.

	CONS (*n* = 7)		*Escherichia coli* (*n* = 88)		*Enterococcus* sp. (*n* = 1)		*Klebsiella pneumoniae* (*n* = 29)		*Proteus* sp. (*n* = 2)	
Antibiotics	S	R	S	R	S	R	S	R	S	R
GEN	1 (100.0)	0 (0.0)	15 (51.7)	14 (48.3)	1 (100)	0 (0.0)	5 (62.5)	3 (37.5)	—	—
CIP	2 (100.0)	0 (0.0)	12 (24.5)	37 (75.5)	0 (0.0)	1 (100.0)	3 (18.8)	13 (81.3)	0 (0.0)	1 (100.0)
SXT	1 (100.0)	0 (0.0)	16 (32.0)	34 (68.0)	—	—	3 (20.0)	12 (80.0)	1 (100.0)	0 (0.0)
CTR			10 (31.3)	22 (68.8)	—	—	2 (20.0)	8 (80.0)	1 (50.0)	1 (50.0)
CTZ	1 (100.0)	0 (0.0)	20 (52.6)	18 (47.4)	—	—	8 (61.5)	5 (38.5)	—	—
MEM			8 (80.0)	2 (20.0)	—	—	4 (100.0)	0 (0.0)	0 (0.0)	1 (100.0)
AMI	1 (100.0)	0 (0.00)	10 (52.6)	9 (47.4)	—	—	6 (60.4)	4 (40.0)	0 (0.0)	1 (100.0)
NAL	0 (0.0)	1 (100.0)	0 (0.0)	12 (100.0)	—	—	3 (75.0)	1 (25.0)	—	—
DOX			1 (14.3)	6 (85.7)	—	—	—	—	—	—
AMO			6 (30.0)	14 (70.0)	—	—	4 (33.3)	8 (66.7)	1 (100.0)	0 (0.0)
AZI	2 (66.7)	1 (33.3)	13 (61.9)	8 (38.1)	0 (0.0)	1 (100.0)	6 (85.7)	1 (14.3)	0 (0.0)	1 (100.0)
OFL			0 (0.0)	4 (100.0)	—	—	—	—	1 (100.0)	0 (0.0)
ERY	1 (100.0)	0 (0.0)	2 (66.7)	1 (33.3)	—	—	0 (0.0)	2 (100.0)	—	—
CTX	1 (100.0)	0 (0.0)	12 (48.0)	13 (52.0)	—	—	1 (10.0)	9 (90.0)	—	—
CMP	0 (0.0)	1 (100.0)	11 (50.0)	11 (50.0)	—	—	5 (50.0)	5 (50.0)	—	—
TET	1 (100.0)	0 (0.0)	10 (37.0)	17 (63.0)	0 (0.0)	1 (100.0)	3 (33.3)	6 (66.7)	—	—
VAN			1 (100.0)	—	—	—	—	—	—	—
CRX			7 (38.9)	11 (61.1)	—	—	2 (22.2)	7 (77.8)	—	—
AMP			2 (33.3)	4 (66.7)	—	—	1 (50.0)	1 (50.0)	—	—
MDR		—		+		+		+		+

Abbreviations: AMI, Amikacin; AMO, Amoxicillin; AMP, Ampicillin; AZI, Azithromycin; CIP, Ciprofloxacin; CMP, Chloramphenicol; CRX, Cefuroxime; CTR, Ceftriaxone; CTX, Cefotaxime; CTZ, Ceftazidime; DOX, Doxycycline; ERY, Erythromycin; GEN, Gentamicin; MDR, multidrug‐resistant; MEM, Meropenem; NAL, Nalidixic Acid; OFL, Ofloxacin; SXT, Trimethoprim/Sulfamethoxazole; TET, Tetracycline; VAN, Vancomycin.

The single *Providencia* sp. isolated in this study displayed high resistance against CIP (100.0%), while no resistance was observed against CTZ (0.0%), MEM (0.0%), and AZT (0.0%). Among the 12 *P. aeruginosa* isolates, significant resistance was observed against NAL (100.0%), DOX (100.0%), AZI (100.0%), CTX (100.0%), CRX (100.0%), SXT (71.4%), CTR (71.4%), and AMI (67.7%). Lower resistance was observed against GEN (28.6%), CIP (40.0%), CTZ (16.7%), MEM (0.0%), AMO (25.0%), and ERY (0.0%). Furthermore, SXT (66.7%), CTZ (100.0%), and TET (87.5%) showed no susceptibility against *S. aureus*. Among the four *S. epidermidis* isolates, high resistance was noted against CIP (100.0%), AZI (100.0%), AMP (100.0%), and TET (50.0%). However, no resistance was observed against GEN (0.0%), SXT (0.0%), and CTZ (0.0%). The single *S. saprophyticus* isolate was 100% resistant to ERY and CTX, but no resistance was observed against SXT (0.0%), AZI (0.0%), and CMP (0.0%) (see Table 5).

**Table 5:** Antibiogram of bacteria isolated in this study.

	*Providencia* sp. (*n* = 1)		*Pseudomonas aeruginosa* (*n* = 12)		*Staphylococcus aureus* (*n* = 17)		*Staphylococcus epidermidis* (*n* = 4)		*Staphylococcus saprophyticus* (*n* = 1)	
Antibiotics	S	R	S	R	S	R	S	R	S	R
GEN	—	—	5 (71.4)	2 (28.6)	4 (57.1)	3 (42.9)	2 (100.0)	0 (0.0)	—	—
CIP	0 (0.0)	1 (100.0)	6 (60.0)	4 (40.0)	5 (55.6)	4 (44.4)	0 (0.0)	1 (100.0)	—	—
SXT	—	—	2 (28.6)	5 (71.4)	4 (33.3)	8 (66.7)	2 (100.0)	0 (0.0)	1 (100.0)	0 (0.0)
CTR	—	—	2 (28.6)	5 (71.4)	—	—	—	—	—	—
CTZ	1 (100.0)	0 (0.0)	5 (83.3)	1 (16.7)	0 (0.0)	2 (100.0)	1 (100.0)	0 (0.0)	—	—
MEM	1 (100.0)	0 (0.0)	3 (100.0)	0 (0.0)	—	—	—	—	—	—
AMI	—	—	1 (33.3)	2 (67.7)	—	—	—	—	—	—
NAL	—	—	0 (0.0)	1 (100.0)	—	—	—	—	—	—
DOX	—	—	0 (0.0)	1 (100.0)	2 (66.7)	1 (33.3)	—	—	—	—
AMO	—	—	3 (75.0)	1 (25.0)	1 (100.0)	0 (0.0)	—	—	—	—
AZI	1 (100.0)	0 (0.0)	0 (0.0)	1 (100.0)	8 (72.7)	3 (27.3)	0 (0.0)	1 (100.0)	1 (100.0)	0 (0.0)
OFL	—	—	—	—	2 (66.7)	1 (33.3)	—	—	—	—
ERY	—	—	1 (100.0)	0 (0.0)	7 (70.0)	3 (30.0)	—	—	0 (0.0)	1 (100.0)
CTX	—	—	0 (0.0)	3 (100.0)	—	—	—	—	0 (0.0)	1 (100.0)
CMP	—	—	—	—	2 (50.0)	2 (50.0)	—	—	1 (100.0)	0 (0.0)
TET	—	—	—	—	1 (12.5)	7 (87.5)	1 (50.0)	1 (50.0)	—	—
VAN	—	—	—	—	—	—	—	—	—	—
CRX	—	—	0 (0.0)	1 (100.0)	—	—	—	—	—	—
AMP	—	—	—	—	—	—	0 (0.0)	1 (100.0)	—	—
MDR		—		+		+		+		—

Abbreviations: AMI, Amikacin; AMO, Amoxicillin; AMP, Ampicillin; AZI, Azithromycin; CIP, Ciprofloxacin; CMP, Chloramphenicol; CRX, Cefuroxime; CTR, Ceftriaxone; CTX, Cefotaxime; CTZ, Ceftazidime; DOX, Doxycycline; ERY, Erythromycin; GEN, Gentamicin; MDR, multidrug‐resistant; MEM, Meropenem; NAL, Nalidixic Acid; OFL, Ofloxacin; SXT, Trimethoprim/Sulfamethoxazole; TET, Tetracycline; VAN, Vancomycin.

## 4 Discussion

The findings of this study provide important insights into the antibiotic resistance patterns of urinary tract pathogens, highlighting the need for continued monitoring and surveillance of antimicrobial resistance (AMR) in this population. The study analyzed data from 2020 to 2023.

### 4.1 Prevalence of AMR

One notable finding of this study is the high prevalence of antibiotic resistance among urinary tract pathogens. The results revealed that *E. coli* exhibited 100% resistance against NAL and OFL, with varying proportions of resistance observed across all other antibiotics, which is consistent with previous studies in the literature [18, 19]. This high rate of antibiotic resistance raises concerns about the effectiveness of commonly used antibiotics for treating UTIs and highlights the need for prudent antibiotic use and appropriate antibiotic stewardship practices in clinical settings.

Another important finding of this study is the emergence of multidrug resistance among urinary tract pathogens. The study identified *E. coli*, *K. pneumoniae*, *P. aeruginosa*, *Proteus* sp., *S. aureus, Enterococcus* spp., and *S. epidemidis* as resistant to three or more classes of antibiotics, indicating a worrisome trend of multidrug resistance. This finding is in line with global concerns about the spread of MDR bacteria, which pose significant challenges for the management of UTIs and other infectious diseases [20, 21].

The study also found variations in antibiotic resistance patterns among different pathogens and over time. For instance, *K. pneumoniae* showed higher resistance rates to GEN, CIP, and SXT compared to *P. aeruginosa* (Tables 4 and 5), suggesting differences in the mechanisms and dynamics of antibiotic resistance among different urinary tract pathogens. Moreover, the study revealed changes in antibiotic resistance patterns over time, with some pathogens showing increasing resistance rates during the study period. These findings highlight the dynamic nature of antibiotic resistance and the need for ongoing monitoring and surveillance to detect and respond to changes in resistance patterns.

Several factors may contribute to the high rates of antibiotic resistance observed in this study. These factors may include inappropriate antibiotic prescribing practices, overuse of antibiotics, patient noncompliance with antibiotic regimens, and inadequate infection control measures in healthcare settings. Strategies to address these factors may include implementing antimicrobial stewardship programs, promoting appropriate antibiotic use, educating healthcare providers and patients on antibiotic resistance and prudent antibiotic use, and improving infection control practices in healthcare facilities.

*Candida* species accounted for 12% of isolates and were included only when laboratory records indicated significant colony counts (> 10⁵ CFU/mL) and associated urinary symptoms, in line with standard diagnostic criteria for fungal UTIs. However, given the retrospective nature of the study and limited clinical details, the possibility of contamination or colonization cannot be excluded. This limitation should be considered when interpreting the prevalence of fungal UTIs in our findings.

### 4.2 Limitations

There are limitations to this study that should be considered when interpreting the results. First, the study was retrospective in nature and relied on data from medical records, which may be subject to incomplete or inaccurate documentation. Second, the study was conducted in a specific population and setting, which may limit the generalizability of the findings to other populations or settings. Third, the study only focused on antibiotic resistance patterns and did not assess other factors that may contribute to the development of antibiotic resistance, such as antibiotic usage patterns, patient demographics, or environmental factors. We were also unable to perform the year‐by‐year trend analysis. Additionally, important confounding variables, such as prior antibiotic exposure, the presence of urinary catheters, and comorbid conditions (especially T2DM), could not be adequately assessed due to missing or incomplete clinical data. These factors are known to influence both the occurrence of UTIs and the emergence of resistant organisms, and their absence may have affected the interpretation of our findings.

## 5 Conclusion

This study provides valuable insights into the antibiotic resistance patterns of urinary tract pathogens. The findings highlight the need for continued monitoring and surveillance of AMR, prudent antibiotic use, and appropriate infection control practices in clinical settings. Further research is warranted to better understand the factors contributing to antibiotic resistance and to develop effective strategies to mitigate the spread of antibiotic‐resistant bacteria in UTIs and other healthcare settings.

## Author Contributions

**Solomon Wireko:** conceptualization, investigation, funding acquisition, writing – original draft, methodology, validation, visualization, writing – review and editing, project administration, supervision, resources, data curation. **Gifty Ngmaakan:** investigation, writing – original draft, methodology, validation, visualization, writing – review and editing, software, data curation, resources. **Doreen Dwomoh:** investigation, writing – original draft, methodology, validation, visualization, writing – review and editing, software, data curation, resources. **Charlotte Hansen:** conceptualization, investigation, methodology, validation, visualization, writing – review and editing, project administration, supervision, resources. **Sampson Nartey:** investigation, writing – original draft, methodology, validation, visualization, writing – review and editing, project administration, supervision, resources. **Ebenezer Senu:** validation, visualization, writing – review and editing, software, formal analysis, data curation, resources, writing – original draft.

## Conflicts of Interest

The authors declare no conflicts of interest.

## Transparency Statement

The lead author, Solomon Wireko, affirms that this manuscript is an honest, accurate, and transparent account of the study being reported; that no important aspects of the study have been omitted; and that any discrepancies from the study as planned (and, if relevant, registered) have been explained.

## Data Availability

All the data obtained and analyzed are included in this manuscript. The data that support the findings of this study are available from the corresponding author upon reasonable request.
